# Viable Group A Streptococci in Macrophages during Acute Soft Tissue Infection

**DOI:** 10.1371/journal.pmed.0030053

**Published:** 2006-01-17

**Authors:** Pontus Thulin, Linda Johansson, Donald E Low, Bing S Gan, Malak Kotb, Allison McGeer, Anna Norrby-Teglund

**Affiliations:** 1Center for Infectious Medicine, Karolinska Institute, Department of Medicine, Karolinska University Hospital, Stockholm, Sweden; **2**Department of Microbiology, Mount Sinai Hospital, University of Toronto, Toronto, Ontario, Canada; **3**Department of Surgery, University of Western Ontario, London, Ontario, Canada; **4**Department of Pharmacology/Toxicology, University of Western Ontario, London, Ontario, Canada; **5**Department of Medical Biophysics, University of Western Ontario, London, Ontario, Canada; **6**Department of Surgery, University of Tennessee, Memphis, Tennessee, United States of America; **7**Department of Microbiology, University of Tennessee, Memphis, Tennessee, United States of America; **8**Department of Immunology, University of Tennessee, Memphis, Tennessee, United States of America; **9**Veterans Affairs Medical Center, Research Service, Memphis, Tennessee, United States of America; University of SussexUnited Kingdom

## Abstract

**Background:**

Group A streptococcal severe soft tissue infections, such as necrotizing fasciitis, are rapidly progressive infections associated with high mortality. Group A streptococcus is typically considered an extracellular pathogen, but has been shown to reside intracellularly in host cells.

**Methods and Findings:**

We characterized in vivo interactions between group A streptococci (GAS) and cells involved in innate immune responses, using human biopsies (*n* = 70) collected from 17 patients with soft tissue infections. Immunostaining and in situ image analysis revealed high amounts of bacteria in the biopsies, even in those collected after prolonged antibiotic therapy. Viability of the streptococci was assessed by use of a bacterial viability stain, which demonstrated viable bacteria in 74% of the biopsies. GAS were present both extracellularly and intracellularly within phagocytic cells, primarily within macrophages. Intracellular GAS were predominantly noted in biopsies from newly involved tissue characterized by lower inflammation and bacterial load, whereas purely extracellular GAS or a combination of intra- and extracellular GAS dominated in severely inflamed tissue. The latter tissue was also associated with a significantly increased amount of the cysteine protease streptococcal pyrogenic exotoxin SpeB. In vitro studies confirmed that macrophages serve as reservoirs for viable GAS, and infection with a *spe*B-deletion mutant produced significantly lower frequencies of cells with viable GAS following infection as compared to the wild-type bacteria.

**Conclusions:**

This is the first study to demonstrate that GAS survive intracellularly in macrophages during acute invasive infections. This intracellular presence may have evolved as a mechanism to avoid antibiotic eradication, which may explain our finding that high bacterial load is present even in tissue collected after prolonged intravenous antibiotic therapy. This new insight into the pathogenesis of streptococcal soft tissue infections highlights a need for alternative therapeutic strategies.

## Abstract 2

This study shows that that group A streptococci survive intracellularly in macrophages during acute invasive infections; the streptococcal pyrogenic exotoxin SpeB may have a role in this survival.

## Introduction

Severe invasive group A streptococcal diseases, including streptococcal toxic shock syndrome (STSS) and necrotizing fasciitis (NF), are rapidly progressive invasive diseases associated with significant morbidity and mortality [1,2]. There is evidence that streptococcal superantigens are important inducers of potent inflammatory responses that contribute to these severe invasive manifestations [3–5]. To date at least 11 distinct superantigens expressed by GAS have been identified, all of which are potent inducers of proinflammatory responses [5,6]. The finding that the human leukocyte antigen class II haplotype of the individual determines the magnitude of the superantigen-induced cytokine response and subsequently the severity of invasive group A streptococcal infection was a major breakthrough in the understanding of the pathogenesis of these infections [7]. More important, the study revealed distinct risk and protective human leukocyte antigen class II associations, depending on whether the patient had STSS or NF. This strongly supports the notion that STSS and NF are distinct clinical entities and that the pathogenic mechanisms leading to each disease may be, at least partly, different.

Although typically considered an extracellular organism, viable GAS have been found intracellularly in epithelial and endothelial cells [8–11] and in neutrophils [12]. Several factors have been suggested to play a role in the internalization process. Among the most well-characterized ones are the fibronectin-binding proteins [13]. Also secreted factors, such as streptolysin O [14,15] and the cysteine protease (i.e., SpeB) [16,17], have been suggested to be involved, but the definite mechanisms and factors involved in the internalization event remain to be elucidated as is evident by the many contradicting reports on this topic (reviewed in [18]).

SpeB is a potent protease that can cleave numerous important human proteins, including extracellular matrix proteins, immunoglobulin, IL1β precursor, and LL37 [19–22]. However, it may also degrade several important streptococcal virulence factors, which results in attenuation of certain virulence properties [23,24]. Rasmussen and Björck [25] suggested that these proteolytic events are tightly regulated both temporally and spatially and differ, depending on the stage of infection. This could explain the varying results from in vivo studies in experimental models and patients' cohorts, some of which implicate SpeB in the disease process [17,26–29], whereas others show an attenuation of virulence if SpeB is expressed [30–32]. Another plausible explanation is that the defined role of SpeB may vary depending on the site of infection, for example, systemic bacteremia versus tissue infection.

We aimed to investigate the pathogenic mechanisms involved in acute group A streptococcal soft tissue infections. The study is based largely on clinically well-defined human tissue biopsy material, consisting of biopsies from areas more or less inflamed and obtained from the same patient, thereby allowing for conclusions regarding the relationship between local disease severity and in vivo expression of host and bacterial factors without bias from infecting strain or specific host properties. We have previously utilized this material to visualize and quantify in vivo host–pathogen interplay with special focus on superantigens and cytokine expression profiles [4]. We extended these analyses to characterize the interaction of GAS with cells of the innate immune system.

## Methods

### Patient Material

The study is based on tissue biopsies (*n* = 70) collected from 17 patients with various soft tissue infections, including NF (*n* = 14), myositis (*n* = 1), and cellulitis (*n* = 2), caused by GAS of varying serotypes, including M1T1, M3T3, M6T6, M28T28, and M4. The patients were identified through active surveillance in Ontario, Canada, during 1995–1997. All patients received intravenous clindamycin in combination with a β-lactam antibiotic at admission. The biopsy material was collected and stored as described in our previous report [4]. Biopsies, including skin, subcutaneous tissue, muscle, or fascia, were collected at surgical procedures performed on different days, ranging from 1–20 d after diagnosis. In several patients (*n* = 11) biopsies of different clinical grades were collected at the same time point. The tissue from which biopsies were taken was graded, based on a clinical assessment made by one of the investigators (DEL) at the time of sampling, according to the following definition. Clinical grade 1 = normal tissue, no evidence of inflammation (*n* = 11). Grade 2 = inflamed tissue (erythema and edema), including 2a = cellulitis (inflammation of skin or soft tissue, but excluding fascia and muscle [*n* = 28]); 2b = fasciitis (inflammation of skin or soft tissue and fascia [*n* = 12]); and 2c = NF (necrotic fascia [*n* = 19]). This study was approved by the Human Subjects Review Committee of the University of Toronto, and informed consent was obtained from all patients. Clinical evaluation, classification of patients, and cell marker/bacterial antigen analyses were performed independently and blinded by different investigators and at different hospitals.

### Immunostaining

The following antibodies specific for human cell markers were used at predetermined optimal dilutions ranging from 1:10–1:400: anti-CD68 (EBM11, murine IgG1; Dako, Carpinteria, California, United States), anti-CD3 (murine IgG1; Becton-Dickinson, Palo Alto, California, United States), anti-CD1a (mouse IgG2a; Dako), antineutrophil elastase (NP57, murine IgG1; Dako). GAS were identified by use of a polyclonal rabbit antiserum specific for the Lancefield group A carbohydrate (diluted 1:10,000; Difco, Detroit, Michigan, United States). In agreement with our previous report [4], the stainings could be divided into high and low amounts of GAS by use of a cutoff image analysis value of five. In vivo expression of SpeB and SpeF was determined by use of polyclonal rabbit antisera raised against native SpeB (Toxin Technology, Sarasota, Florida, United States) or against recombinant SpeF (produced in the laboratory of MK). Anti-SpeB and anti-SpeF sera were used at a dilution of 1:2,000 and 1:10,000, respectively. The specificity of the polyclonal anti-SpeB antiserum was tested by preincubation of the antiserum with purified SpeB. Preincubation with a 10-fold excess of purified SpeB effectively blocked staining for the antigen in the tissue sections. Irrelevant isotype-specific murine antibodies (Vector Laboratories, Burlingame, California, United States) and preimmune rabbit sera at appropriate dilutions were used to control for nonspecific staining reactions. Biotinylated secondary antibodies included goat-anti-mouse IgG1, goat-anti-mouse IgG2a (diluted 1:300; CalTag Laboratories, Burlingame, California, United States), and goat-anti-rabbit IgG (diluted 1:500; Vector Laboratories).

### Immunohistochemical Staining of Tissue Biopsies

The biopsies were cryosectioned, fixed, stained, and analyzed by image analysis as previously described [4]. The staining method was modified to include an initial blocking step with 20% FCS in BSS-saponin for 30 min at room temperature followed by 1% normal goat serum for 30 min. GAS and SpeF antibodies were diluted in Earl's balanced salt solution containing saponin and 0.02% NaN_3_. Addition of 10% FCS was required together with the SpeB antisera to minimize unspecific staining. Regardless of the size of the biopsies, which varied substantially, the whole section was analyzed by acquired computerized image analysis (ACIA), yielding an analyzed cell area (defined by the blue hematoxylin counterstaining) ranging from 0.4 × 10^5^–1.6 × 10^7^ μm^2^. The results are presented as ACIA values, which equal percentage positively stained area × mean intensity of positive staining. The majority of negative controls, i.e., biopsies stained with isotypic control antibodies, were completely negative; however, in case of any background staining, the sections were subjected to ACIA analyses and the value subtracted from the sample value.

### Fluorescent Staining and Confocal Microscopy

Single and double immunostainings were conducted for GAS and SpeB, GAS and CD68, and GAS and neutrophil elastase, respectively. The tissue biopsies were sectioned, fixed, and permeabilized as described above. Tissue sections were initially blocked with 2% FCS in PBS-saponin for 30 min followed by avidin and biotin blocking (Vector Laboratories) for 15 min each. This was followed by blocking with PBS-saponin containing 0.1% BSA-c (Aurion, Wageningen, The Netherlands). GAS-, SpeB-, CD68-, secondary antibodies, and also fluorophores were all diluted in PBS-saponin-BSA-c. Primary antibodies were incubated overnight. Antibodies against SpeB were conjugated with EZ-Link NHS-LC-biotin (Pierce Biotechnology, Rockford, Illinois, United States) according to the manufacturer's manual. GAS and CD68 stainings required a 30-min step with biotinylated secondary antibodies. After incubation with the biotinylated secondary antibody, streptavidin-conjugated fluorophores (Alexa Fluor 488 diluted 1:600, and Alexa Fluor 546 diluted 1:500; Molecular Probes, Eugene, Oregon, United States) were added and incubated for 30 min in the dark. Both the antisera and the secondary biotinylated antibodies were used at optimized concentrations. Determination of bacterial viability was achieved through staining of 16-μm thick biopsy sections with a live/dead Baclight bacterial viability kit (Molecular Probes). For evaluation, a Leica confocal scanner TCS SP II was used, coupled to a Leica DMR microscope (Leica, Wetzlar, Germany). We used a conservative evaluation of the biopsies, including the criterion of the presence of a significant number of bacteria (with a bright green fluorescence, intact morphology, and correct size) in the biopsy in order to be classified as positive for viable bacteria. The specificity of the viability kit was validated by using antibiotic- and heat-killed streptococci, as well as a dose-response experiment. In the latter experiment three different doses (corresponding to a multiplicity of infection 10, 5, and 1) of live *Streptococcus pyogenes* was used for infection of macrophages, and intracellular survival assessed by the viability stain was compared to bacterial growth on blood agar plates following cell lysis. The two methods showed a good concordance.

### In Vitro Infection of Monocytes and Macrophages

GAS isolates 8157, 5448, and the 5448-derived SpeB in frame allelic-exchange knockout 5448ΔSpeB (generated by Dr.V. Nizet, La Jolla [33]), were used for in vitro infection. The strains were of M1T1 serotype and isolated from patients with STSS, including one patient from whom biopsies were obtained and included in the present biopsy material. The isolates were grown at 37 °C to stationary phase in Todd-Hewitt broth (Difco) supplemented with 1.5% yeast extract (Difco), washed with PBS, and diluted to the required inoculum (1–2.5:1, bacteria to cell ratio) as determined by viable counts on sheep blood agar plates. Peripheral blood mononuclear cells were isolated from buffy coats or from heparinized venous blood by Ficoll-Hypaque gradient centrifugation. Monocytes were enriched through plastic adherence for 90 min and either used directly after washing, or allowed to differentiate into macrophages through cell culture for 7–10 d in RPMI 1640 medium (Gibco, Grand Island, New York, United States) supplemented with 10% FCS. GAS isolates were added to washed cells and infection allowed to proceed for 120 min at 37 °C, 5% CO_2_, after which 125 μg/ml gentamicin (Gibco) and 2.5 μg/ml Pc-G were added for 45 min, followed by extensive washing and incubation in RPMI supplemented with 10% FCS and Pc-G at 1 μg/ml. Intracellular bacteria were assessed after various time points by use of the live/dead Baclight bacterial viability kit (Molecular Probes) in 0.05% saponin to permeabilize cell membranes. Confocal microscopy was used to determine the frequency of monocytes/macrophages harboring dead and/or viable bacteria. More than three viable GAS per cell were required in order for a cell to be assessed as harboring viable GAS. Bacterial viability was confirmed at the early time points by cell lysis in Milli-Q containing 0.025% Triton-X and subsequent determination of viable counts on blood agar plates. At the later time points, bacterial growth was assessed in antibiotic-free medium, as described below. Growth curves of 5448 wild type and ΔSpeB were similar.

### Bacterial Resurgence into Medium after Antibiotic Removal

Macrophages were infected with strains 8157 or 5448. At 2 h postinfection, gentamicin (125 μg/ml) and penicillin-G (2.5 μg/ml) were added for 45 min to kill extracellular bacteria. The cells were then washed three times with PBS and incubated in RPMI containing 10% FCS and 1 μg/ml of penicillin-G. At 12 h postinfection, cells were washed three times with PBS, and fresh antibiotic-free RPMI containing 10% FCS was added. At different time points, samples were taken from the medium and plated on blood agar plates. Plates were incubated overnight at 37 °C, and then colonies were counted.

### Statistical Evaluation

Comparisons between groups were performed using a Mann-Whitney test, Kruskal-Wallis median test, or—in the case of categorical values—Fisher's exact test. Deming regression (i.e., model II linear regression) was used to examine the relationship between variables. SpeB, SpeF, and GAS levels were transformed with the square root in order to normalize the distribution of these variables. *p*-Values of less than .05 were considered significant.

## Results

### High Bacterial Load and Infiltration of Phagocytic Cells at the Local Site of Infection

In agreement with our previous study [4], all biopsies (*n* = 70) in our expanded clinical material contained varying amounts of bacteria, with the highest levels noted in biopsies of higher clinical grade, i.e., severely involved tissue with high inflammation (*p* < 0.01; Figure 1A). Strikingly, biopsies obtained more than 3 d after diagnosis of infection and initiation of intravenous antibiotic (*n* = 24 biopsies) still contained GAS, and 50% of those had high amounts of the bacteria (Figure 1A).

**Figure 1:**
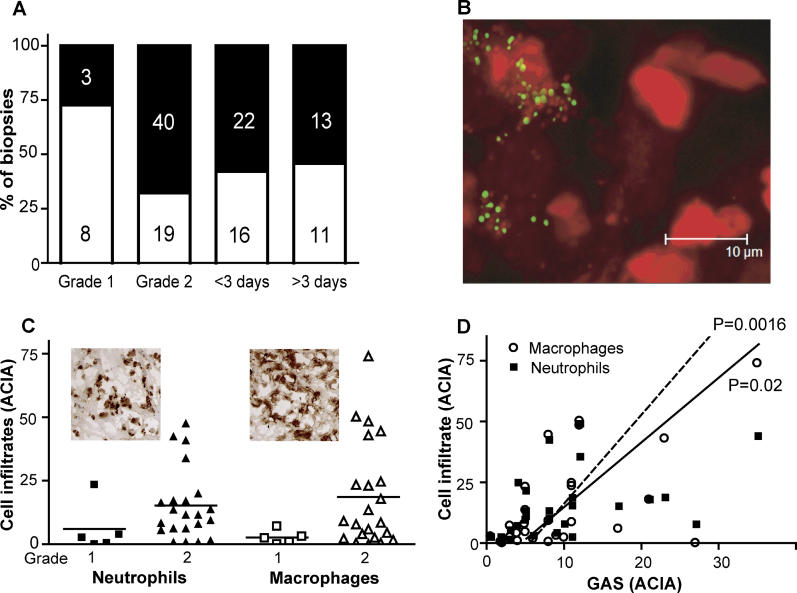
High Bacterial Load and Infiltration of Phagocytic Cells in Tissue Biopsies Tissue biopsies of clinical grades 1 or 2 (for definitions, see Methods) were stained for the presence of bacteria, macrophages (CD68-positive cells), and neutrophils (neutrophil elastase-positive cells). The stained biopsies were evaluated by ACIAs, and the ACIA value is shown in the figure; for details, see Methods. (A) Percentage of biopsies with low or high bacterial load (indicated by white and black bars, respectively) in biopsies divided according to their clinical grade (1 or 2) or by time of collection after diagnosis. Numerals within the bars denote number of biopsies. Low or high bacterial is defined by the threshold ACIA value of ≥5, as previously described [4]. The viability of GAS in the tissue was assessed by use of a bacterial viability stain that stains viable bacteria green and dead bacteria red, as shown in (B). Note that the viable bacteria are mainly found associated with cells indicated by their red cell nuclei. (C) Degree of neutrophil and macrophage infiltration in biopsies of varying clinical grade. The insets show representative immunohistochemical stainings. (D) Amount of neutrophils (squares) and macrophages (dashed line and circles) in relation to bacterial load, i.e., GAS ACIA. Significant correlation as determined by Deming regression is indicated by *p*-values.

The fact that intact streptococci of normal size (0.8 μm) and, as presented below, local expression of exotoxins are present at the site of infection strongly implied that the bacteria within the tissue were viable. However, bacterial culture before snap freezing had only been performed on one biopsy. This biopsy, which had been collected 7 d after diagnosis, was found positive for GAS growth. To confirm the viability of the bacteria within the frozen tissue, 19 biopsies were analyzed using a bacterial viability stain that allows for distinction of viable and dead bacteria within the tissue. Conservative evaluation of these stainings, with respect to morphology and staining intensity, demonstrated viable bacteria in the majority of biopsies (74%), and most viable bacteria were found to be associated with host cells (Figure 1B).

To examine whether the high bacterial load could be due to lack of infiltrating phagocytic cells, as was recently suggested [34], the degree of phagocytic cell infiltration in these biopsies was determined. The majority of biopsies (89%) contained both neutrophils (i.e., neutrophil elastase-positive) and macrophages (i.e., CD68-positive, CD1a-negative cells), with somewhat higher levels of macrophages (Figure 1C). The phagocytic cellular infiltration was generally higher in biopsies of clinical grade 2—i.e., severely involved tissue (Figure 1C)—and correlated significantly with bacterial load (*p* < 0.02; Figure 1D). Essentially no dendritic cells could be detected in the biopsies, as evident by the lack of CD1a positive cells.

### Intra- and Extracellular Localization of GAS in the Tissue

Immunohistochemical staining for GAS revealed three distinct staining patterns that were highly reproducible in each biopsy: intracellular GAS defined by a cytoplasmic staining pattern (Figure 2A), a combination of intra- and extracellular bacteria (Figure 2B), and extracellular cocci in the tissue (Figure 2C). Surprisingly, 43% of the biopsies showed a pattern of purely intracellular bacteria. The cellular localizations were confirmed by immunostaining and confocal microscopic analyses (Figure 2D). In order to identify the host cells that harbor GAS, dual immunostainings of GAS and various cell markers, including CD1a, CD3, CD68, and neutrophil elastase, revealed that intact GAS were found predominantly in macrophages, i.e., CD68-positive, CD1a-negative cells, although GAS could also be detected within neutrophils occasionally.

**Figure 2:**
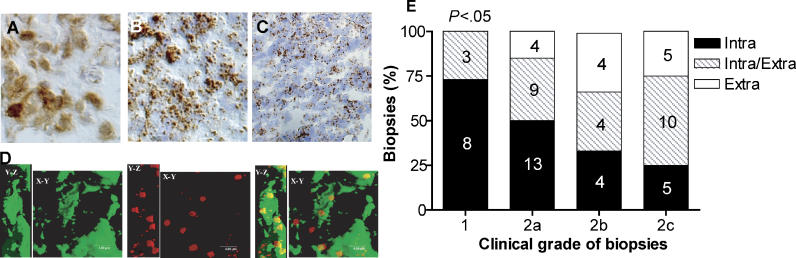
GAS Were Localized Extracellularly or Intracellularly in Phagocytic Cells in the Tissue Biopsies from patients with tissue infections (*n* = 70) caused by GAS were cryosectioned and immunostained for GAS. Immunohistochemical staining revealed three main staining patterns: (A) cytoplasmic, intracellular staining; (B) a combination of intra- and extracellular staining; and (C) extracellular staining. (D) Confocal microscopy of GAS (Alexa 546, red) and CD68-positive cells (Alexa 488, green) in the tissue. The figure shows a simulated fluorescence process projection of a sequential scan. The Y–Z area equals the vertical plane. Intracellular streptococci are shown in yellow. (E) Bacterial localization in relation to clinical grade. Tissue biopsies from patients with tissue infections caused by GAS were classified according to clinical grade (for definitions, see Methods). Numerals within bars denote number of biopsies. Statistical difference between groups was analyzed using Fisher's exact test.

Intracellular bacterial localization was found to be significantly more common in biopsies of clinical grade 1, i.e. newly involved tissue with low inflammation and low bacterial load, than in biopsies of higher clinical grade and excessive inflammation (*p* < 0.05; Figure 2E). The amount of infiltrating phagocytic cells, bacterial load, or their cellular localization did not correlate significantly to time of tissue collection in relation to disease onset or to the type of tissue collected (unpublished data).

### In Vivo Expression of SpeB Varies Depending on Bacterial Localization

Conflicting data on the role of the streptococcal cysteine protease SpeB in the internalization event have been published [16–18,35–37]. Our tissue material allowed for a direct assessment of the in vivo expression of SpeB during the acute infection. SpeB was detected in most biopsies (82%), and the amount of SpeB showed a positive correlation to the bacterial load (*p* = 0.0006; Deming regression; unpublished data). More important, significantly higher amounts of SpeB was seen in biopsies where GAS were localized both intra- and extracellularly, as compared to biopsies where the bacteria were found either intra- or extracellularly (*p* = 0.03; Figure 3A). This expression pattern seems to be unique to SpeB, since SpeF was found in equal amounts in biopsies with purely intracellular GAS or those with a combination of intra- and extracellular GAS. A potential bias due to treatment could be excluded since a subanalysis, which involved only biopsies obtained at day 1, showed a similar correlation between GAS localization and the differential expression of SpeB and SpeF (unpublished data).

**Figure 3:**
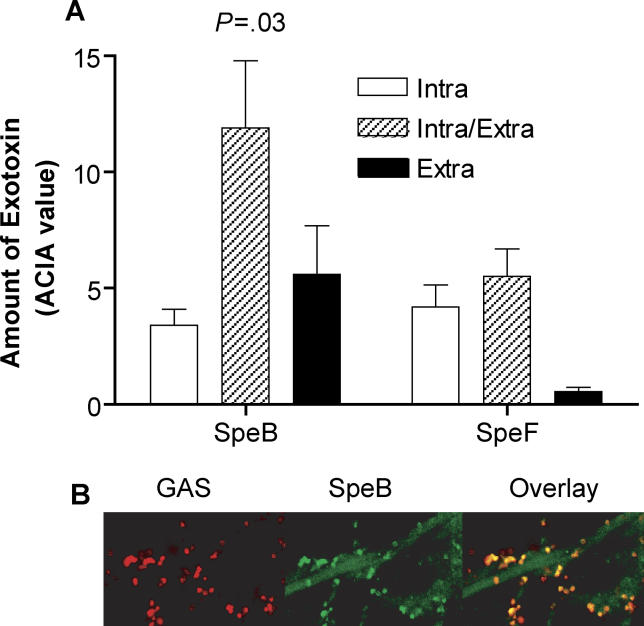
Amount of Spe in Tissue Biopsies with Different Bacterial Localization Tissue biopsies were immunohistochemically stained for GAS, SpeB, and SpeF, and the stainings were quantified by ACIA. (A) Amount of SpeB and SpeF in biopsies with different bacterial localization (*n* = 65 and 67, respectively); Intra, intracellular; Intra/Extra, intra and extracellular; and Extra, extracellular. Bars show mean ± SEM. Statistically significant differences were estimated by a Kruskal-Wallis median test. (B) SpeB attached to the bacterial cell surface in a representative tissue biopsy. Confocal microscopy of immunostainings of GAS (Alexa 546, red) and SpeB (Alexa 488, green). Colocalized staining is shown in yellow.

### Evidence for the Presence of Secreted and Cell-Attached Forms of SpeB In Vivo

Immunostaining for both GAS cell-wall carbohydrates and SpeB in the tissue biopsies revealed that SpeB could be found secreted in large areas of the tissue, or colocalized to the group A carbohydrates, i.e., attached to the bacterial surface (Figure 3B). In all SpeB-positive biopsies, the secreted form of SpeB could be detected, whereas the surface attached form was only found in ten biopsies obtained from six patients infected with GAS isolates of varying serotypes. Nine of these ten biopsies were of severely involved tissue, and seven had a high bacterial load with GAS located both intra- and extracellularly. The secreted and surface-attached forms were found in distinct areas of these biopsies, where the attached form was predominantly localized to areas with higher cellular density (i.e., areas with strong hematoxylin nuclear counterstaining).

### In Vitro Studies Confirm the Presence of Intracellular Viable GAS in Macrophages

To confirm our in vivo findings, the internalization frequency and intracellular survival of clinical GAS isolates was analyzed in an in vitro cell culture system, using freshly isolated human monocytes or monocyte-derived macrophages. First we tested whether the internalization frequency of GAS was affected by the presence of human serum, but no such effect was found as equal frequencies of cells with viable GAS were found in cultures with either human serum or fetal calf serum (unpublished data). The experiments were therefore conducted in the absence of human serum to prevent an influence of opsonizing antibodies, which is clinically relevant since studies have shown that patients developing severe invasive infection lack, or have very low levels of, such antibodies [38,39]. After an initial infection time of 2 h followed by killing of extracellular bacteria by antibiotics, both monocytes and macrophages were found to contain large numbers of viable bacteria (Figure 4A and 4B). The two clinical invasive GAS strains used resulted in similar frequencies of infected cells. Approximately 30%–60% of the cells were found to harbor viable intracellular bacteria at both 4 and 12 h postinfection (Figure 4A), and intracellular viable bacteria could be detected for up to at least 20 h (unpublished data). Removal of extracellular antibiotics 12 h postinfection led to a rapid resurgence of bacteria in the culture medium after a lag time of about 2 h. An initial 10-fold increase per hour could be observed for both strains (Figure 4C), which far exceeded the growth rate of the strains in control experiments without cells. Although the majority of the intracellular bacteria were viable, a significant number of dead bacteria were also noted intracellularly (Figure 4B). This is likely the result of an efficient phagocytic killing; however, some of the dead bacteria may also represent phagocytosed adherent bacteria that were killed by the extracellular antibiotic.

**Figure 4:**
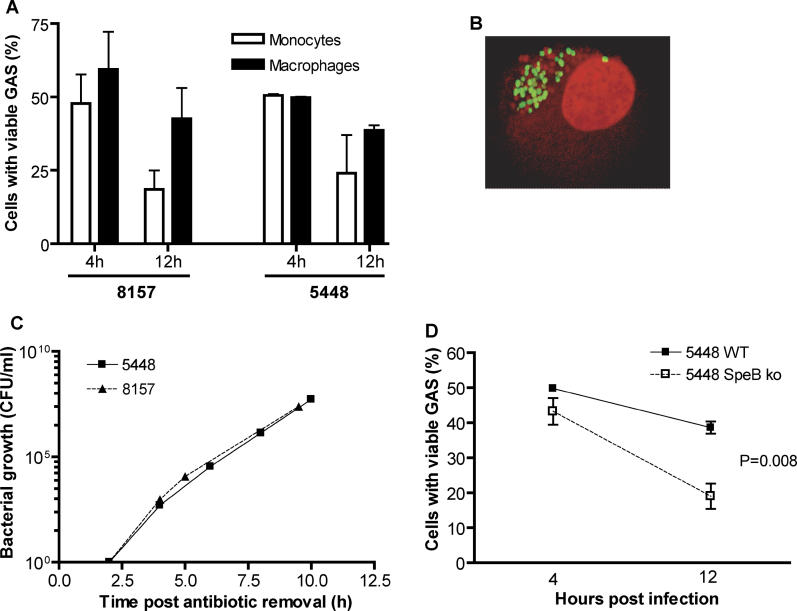
GAS Survive Intracellularly in Monocytes and Macrophages A) Frequency of cells containing viable intracellular bacteria 4 and 12 h postinfection with strains 8157 and 5448, respectively. Bars denote mean ± SEM of four or five experiments performed using monocytes or macrophages, respectively. (B) A confocal image of a representative macrophage harboring intracellular GAS (viable in green and dead in red) as assessed by staining with a bacterial viability kit. (C) Resurgence of intracellular bacteria (8157 and 5448) in the medium. At 12 h postinfection, antibiotic-free medium was added, and extracellular growth was monitored in the medium over time. The figure shows one representative out of four experiments performed. Control experiments lacking live human cells failed to support growth after antibiotic removal (unpublished data). (D) Comparison of frequencies of cells harboring viable GAS after infection with either 5448 wild-type (WT) strain or its isogenic ΔSpeB knockout (SpeB ko). Infected cells were stained with a live/dead BacLight bacterial viability kit, at 4 and 12 h postinfection, and the frequencies of cells containing viable bacteria in relation to the total number of cells were determined. Mean ± SEM of five experiments performed is shown. Significant difference in survival over time was determined by a Mann-Whitney test, and the *p*-value is indicated.

To analyze the contribution of SpeB in the internalization process, we compared the wild-type clinical isolate 5448 to its isogenic *spe*B gene-deletion mutant. The frequencies of cells with viable GAS were similar at 4 h postinfection; however, at 12 h postinfection, significantly lower frequencies of cells with viable GAS were seen following infection with the *spe*B mutant as compared to the wild type (*p* = 0.008; Figure 4D).

## Discussion

This study demonstrated that GAS can reside intracellularly in host phagocytic cells at the local site of tissue infection during the acute phase. More important, the localization of GAS varied depending on severity of tissue infection, and the intracellular localization was most prevalent in noninflamed tissue, which also demonstrated low bacterial load. Both neutrophils and macrophages were found to harbor intracellular GAS, but intact streptococci were more commonly observed in macrophages. An intracellular reservoir of GAS in neutrophils and epithelial cells has previously been shown through in vitro studies and experimental murine models [8,9,40]. Furthermore, the study by Osterlund et al. [10] revealed intracellular GAS present in pharyngeal epithelial and macrophage-like cells of patients with tonsillitis. Here we show, for the first time, that this occurs in vivo during severe invasive GAS soft tissue infections in humans, even while patients are on antibiotic therapy.

The role of macrophages as host cells for intracellular GAS was further confirmed by in vitro cell culture experiments demonstrating a high frequency of monocytes and macrophages harboring viable GAS. More important, the bacteria could survive intracellularly for an extended period of time, and once the antibiotic was removed, a striking extracellular bacterial growth could be observed. This result implies that streptococci residing intracellularly in macrophages represent an important reservoir of bacteria that, once they leave their host cell, are capable of causing continued infection.

Phagocytic cells are believed to have an essential role in the host defense against infections, as was recently shown by Goldmann et al. [41] who reported an important role of macrophages in the early control of GAS infections. Although we found that phagocytes manage to kill some of the bacteria, as evident by the bacterial viability assessment, our data clearly demonstrate that a high proportion of the internalized bacteria remain viable for an extended period of time within the phagocytic cells. The underlying explanation for intracellular killing rather than survival within phagocytes remains to be elucidated, but it seems likely that the entry pathway is involved. This theory is supported by our recent data that addition of polyclonal intravenous immunoglobulins containing opsonizing antibodies significantly improved phagocytic killing of GAS in our cell culture system (LJ, PT, and ANT, unpublished data). Various mechanisms to promote intracellular survival in host cells have recently been suggested, including cytolysin-mediated evasion of lysosomal killing in nonphagocytic cells [14,15] and utilization of host cell caveolae as entry port into epithelial and endothelial cells [11]. The pathway involved in intracellular survival of GAS in macrophages is as yet unknown.

A clinically important finding of this study is that superantigen-producing GAS could be detected in tissue biopsies obtained several days (3–24 d) after the onset of infection, despite ongoing antibiotic therapy (intravenous clindamycin in combination with a β-lactam antibiotic) during this time period. One of the biopsies included in this study was assessed for bacterial growth on a blood agar plate, which revealed growth of bacteria in areas distant to the biopsy, whereas a clear inhibitory zone around the biopsy was seen. Hence, the biopsy contained substances that were toxic to the bacteria. Whether these are antibiotics and/or other bacteriostatic substances, such as host antimicrobial peptides, remains an open issue. However, our data clearly show that despite the presence of these toxic substances within the biopsy, the bacteria still resist killing, potentially through the intracellular persistence in host cells. Antibiotic eradication failure due to an intracellular streptococcal reservoir has previously been reported for recurrent tonsillitis cases [42,43]. The results emphasize that alternate approaches to therapy may be required if we are going to improve the morbidity and mortality associated with severe GAS soft tissue infections.

The present study also demonstrates, for the first time in patients, that SpeB is secreted at the local site during these infections. The toxin was found secreted in large areas of the tissue and, considering the potent proinflammatory and proteolytic activities of SpeB, such high local expression is likely to contribute to the excessive inflammation previously reported for these tissue infections [4]. In addition, the analyses show that the bacterial-attached form of SpeB occurs in vivo, an observation that has previously been reported in vitro [19,44]. In vivo, this phenomenon was observed predominantly in severely involved tissue with a combination of intra- and extracellular bacteria and dense cell infiltration. Taken together with our finding of an intracellular source of streptococci in vivo, these data could imply that bacterial-attached SpeB may contribute to this intracellular reservoir through its strepadhesin activity previously reported by Hytönen [44]. In vitro infection of macrophages with a SpeB mutant confirmed a role for SpeB for intracellular survival within macrophages. Whether this is linked to the adherence, internalization pathway, or to some other mechanism remains to be shown. One intriguing hypothesis is that the bacterial-attached SpeB could promote an alternative entry pathway into the macrophages that would allow the bacteria to avoid phagocytic killing.

Our finding that intracellular bacteria were most commonly found in newly involved tissue with low inflammation and low bacterial load leads us to propose a hypothetical model that internalization can promote the spread of bacteria within the tissue. This is in analog to the fish, and occasionally also human, pathogen *Streptococcus iniae* that uses macrophage-like cells as “Trojan horses” in their natural host [45]. Studies are currently ongoing to explore whether this also occurs in GAS soft tissue infections.

**Patient Summary**

**Background**

One cause of a very severe soft infection called necrotizing fasciitis is a bacterium known as group A streptococcus. This infection can spread very quickly, and it can be fatal. Treatment of this infection often requires more than antibiotics: for example, removal of the infected tissue in surgery. Previous work has shown that these bacteria can live inside cells that would normally kill them.

**Why Was This Study Done?**

The authors wanted to investigate whether an intracellular streptococci persisting in human cells contributes to the development and severity of severe soft tissue infections.

**What Did the Researchers Do and Find?**

They looked at 70 tissue samples taken from 17 people with soft tissue infections. They found that many live bacteria were present both inside and outside of the cells that take up bacteria—particularly one type of cell known as a macrophage—and even when the samples were taken after patients had had a long course of antibiotics. The bacteria were mostly inside cells in samples taken from newly infected and less inflamed tissue, whereas more severely inflamed tissue had bacteria only outside of the cells or both inside and outside. The authors also showed that the severely inflamed tissue had increased amounts of an enzyme called streptococcal pyrogenic exotoxin (SpeB), which destroys some proteins from infected tissue. Experimental infections with bacteria that had this enzyme removed led to lower numbers of cells with living bacteria in them compared with the normal bacteria.

**What Do These Findings Mean?**

It appears that these bacteria can hide inside cells that should kill them and thus be protected from the effects of antibiotics. The enzyme SpeB may play an important role in whether the infection becomes more serious. This study will not affect how patients with this disease are treated at the moment, but may suggest further research that needs to be done. Future studies may need to suggest new ways to get rid of these bacteria.

**Where Can I Get More Information Online?**

MedlinePlus has a Web page of information and other links on necrotizing fasciitis:

http://www.nlm.nih.gov/medlineplus/ency/article/001443.htm

The US Centers for Disease Control and Prevention has a Web page on Group A streptococcus:

http://www.cdc.gov/ncidod/dbmd/diseaseinfo/groupastreptococcal_g.htm

## Abbreviations

ACIA: acquired computerized image analysis
GAS: group A streptococci
NF: necrotizing fasciitis
SEM: standard error of the mean
Spe: streptococcal pyrogenic exotoxin
STSS: streptococcal toxic shock syndrome
